# Is molecular breast imaging suitable for use in UK breast cancer pathways? A qualitative study exploring healthcare professionals’ perspectives

**DOI:** 10.1136/bmjopen-2025-113676

**Published:** 2026-06-03

**Authors:** Helen Elliott, A. Joy Allen, Nerys D Forester, Sara Graziadio, William Stephen Jones, Clare Lendrem, Mark Pearce, Timothy Powell, Alison Bray, Jason Scott

**Affiliations:** 1 Northern Medical Physics and Clinical Engineering, Newcastle Upon Tyne Hospitals NHS Foundation Trust, Newcastle upon Tyne, UK; 2 Newcastle University, Newcastle upon Tyne, England, UK; 3 NIHR Newcastle In Vitro Diagnostics Co-operative, Newcastle upon Tyne, England, UK; 4 Radiology, Newcastle Upon Tyne Hospitals NHS Foundation Trust, Newcastle upon Tyne, England, UK; 5 Newcastle Upon Tyne Hospitals NHS Foundation Trust, Newcastle upon Tyne, UK; 6 Population Health Sciences Institute, Newcastle University, Newcastle upon Tyne, England, UK; 7 Faculty of Health and Wellbeing, Northumbria University, Newcastle upon Tyne, UK

**Keywords:** Early Detection of Cancer, Cancer, NUCLEAR MEDICINE, Breast tumours, QUALITATIVE RESEARCH, Breast imaging

## Abstract

**Abstract:**

**Objectives:**

To explore healthcare professionals’ perspectives on the potential role of molecular breast imaging (MBI) for breast cancer imaging and to inform future clinical study design and implementation.

**Design:**

Qualitative interview study.

**Setting:**

UK National Health Service (NHS) breast screening and diagnostic pathways.

**Participants:**

Purposively sampled stakeholders.

**Method:**

Semistructured interviews with key professional stakeholders explored potential MBI pathways and routes to adoption, including barriers and facilitators. Data were analysed thematically.

**Results:**

22 participants were recruited between January 2020 and October 2021. Barriers to MBI adoption were identified at three levels: scan-related, system-level, and cultural within the screening programme. Overcoming these is likely necessary for implementation. A further theme highlighted the potential for MBI to improve screening in selected patient groups, contingent on addressing these barriers. Specifically, adoption would require advances in next-generation MBI systems, particularly reductions in radiation dose and scan time, alongside prospective clinical studies in UK populations to assess diagnostic accuracy.

**Conclusions:**

Once identified barriers are overcome, participants perceived that MBI could improve screening pathways, particularly for women with dense breast tissue.

**STRENGTHS AND LIMITATIONS OF THIS STUDY**

- The study had a clear and comprehensive sampling strategy using both purposive and snowball sampling with sample size informed by information power.
- The use of remote, online interviewing, adopted due to COVID-19 restrictions, enabled broader geographical reach and increased accessibility, allowing participation from individuals in senior or specialised roles who may have been less able to take part in face-to-face interviews.
- While all participants were provided with the same structured information about molecular breast imaging (MBI), including diagnostic accuracy and example images, it is likely that their understanding of the intervention varied.
- Participants’ perspectives were informed by the diagnostic-accuracy data available in 2021. Subsequently published evidence, including the Density MATTERS Trial, may influence contemporary understanding of MBI performance.
- While dual interviewing enhanced transparency, reflexivity and coverage of topics, the presence of two researchers may have influenced participant openness and had an indirect effect on participant responses.

## Background

 Over 55 000 cases of breast cancer are diagnosed in the UK annually, and over 11 500 women die from the disease.^1^ Cancer screening programmes aim to identify life-threatening tumours early, reduce future treatment costs and decrease mortality. The National Health Service Breast Screening Programme (NHSBSP) includes two initiatives: routine screening for women aged 50–70 three yearly using mammography and screening for women at very high risk of breast cancer, with frequency and methods tailored to individual risk.

The NHSBSP is one of several interconnected breast cancer imaging pathways, including for symptomatic patients, monitoring disease response and post-cancer surveillance. Mammography is the primary imaging test, while targeted ultrasound (USS) is used to guide lesion biopsy. MRI, a more sensitive test, is reserved for groups where it is most effective.

Mammography detects tumours by distinguishing their density from surrounding normal tissue, but this can miss tumours in women with dense breasts.^2^ Recent evidence suggests that women with dense breasts should be offered additional imaging due to mammography’s limited sensitivity and their independently elevated risk of breast cancer.^3 4^ This is reflected in updated European Union (EU) guidelines, which recommend informing women about their breast density and offering additional breast MRI to women with very dense tissue,^5^ as categorised by the Breast Imaging Reporting and Data System (BI-RADS).^6^

Molecular breast imaging (MBI), a nuclear medicine imaging technique, is used in the USA for screening women with dense breasts.^7^ Studies have demonstrated that MBI has superior diagnostic accuracy versus mammography for this group.^8 9^ While this suggests that MBI could have a place in UK breast imaging pathways, no research has yet explored whether its adoption would be acceptable to stakeholders or the factors to be considered for its optimal integration.

This exploratory qualitative study aimed to address this evidence gap by examining UK stakeholders’ perspectives on relevant patient populations, and the barriers and facilitators to adopting MBI as a breast-imaging test. The findings are intended to inform future development, evaluation and implementation of MBI.

## Methods

The work described in this paper shared methodology with a parallel study exploring patient perceptions of MBI.^10^ Deviations from that methodology are described below.

### Participants, sampling, recruitment

Purposive and snowball sampling were used to select participants with experience in breast screening or nuclear medicine within the UK. Inclusion criteria were current or recent experience in these areas; others were excluded. Invitations were sent by email through clinical networks across National Health Service (NHS) breast-screening units and nuclear medicine departments. A pragmatic sample size of 20 participants was chosen after consultation with experienced qualitative researchers and considering information power.^11^ No incentives were offered.

### Data collection and analysis

Qualitative, semistructured, exploratory, single participant interviews were a mixture of face-to-face and online, following written or recorded, informed consent. Interviews, based on an iteratively designed topic guide (included in online supplemental information), focused on consideration of potential new patient pathways incorporating MBI, routes to adoption, and barriers and facilitators. Topic guides were developed and tested in two pilot interviews with breast screening and nuclear medicine specialists. Two interviewers (HE as interview lead and a second researcher to support) were present for all but one interview, improving transparency, reflexivity and topic coverage, though their dual presence may have affected participant openness and indirectly shaped responses.

Participants were shown example MBI images, diagnostic accuracy summaries and key test characteristics, although understanding of this information likely varied despite standardised materials. The structured information about MBI presented to participants reflected the best available evidence at that time, primarily early diagnostic-accuracy studies from single-institution cohorts.

The interviews were conducted by HE, a researcher with 9 years’ experience in NHS-based, patient-facing cancer research, and AB, a clinical scientist with 11 years’ experience implementing novel imaging technologies in NHS pathways. Neither had lived experience of breast cancer or screening. Analysis was supported by JS, an associate professor and chartered psychologist, at the time of the study, with extensive expertise in qualitative health-systems research.

Data were analysed using reflexive thematic analysis; HE undertook the coding, with regular discussion with AB and JS to support analytic rigour. Full methodological details are provided in our previously published paper.^10^

### Patient and public involvement

Patients and members of the public were involved in the design of this study through a patient and public involvement (PPI) panel that met quarterly as part of a wider project. The panel reviewed and provided input into the study aims and the interview topic guide used during data collection. The panel were not directly involved in conducting the interviews or analysing the data due to ethical and regulatory constraints.

## Results

Data collection was undertaken between January 2020 and October 2021. 22 participants were recruited, of which six (27%) were face-to-face and 16 (73%) were online. Interview time ranged from 41 min to 1 hour and 22 min, with a mean of 1 hour and SD of 11 min. Table 1 presents participant demographics. Participants were recruited nationally within the UK and included senior members of NHS England, Cancer Alliances and professorial level clinical academics, staff at leading national cancer charities and relevant specialist advisory bodies within government.

**Table 1:** Participant demographics

	Number of staff participants (percentage of sample)
Total	22 (100%)
Job title	
Consultant breast radiologist	8 (36%)
Policy manager	5 (23%)
Senior breast radiographer	2 (9%)
Specialist breast care nurse	2 (9%)
Consultant nuclear medicine radiologist	1 (5%)
Consultant clinical oncologist	1 (5%)
Consultant clinical scientist in nuclear medicine	1 (5%)
Consultant clinical geneticist	1 (5%)
Consultant oncoplastic breast surgeon	1 (5%)
Gender	
Female	15 (68%)
Male	7 (32%)
Length of time in role	
Less than 10 years	11 (50%)
More than 10 years	8 (36%)
Unknown	3 (14%)
Area of work	
Breast	15 (68%)
Nuclear medicine	3 (14%)
Other (third sector, genetics)	4 (18%)
Location	
North [additional national role]	12 [1] (55%)
South [additional national role]	6 [6] (27%)
National	4 (18%)

**Figure 1:**
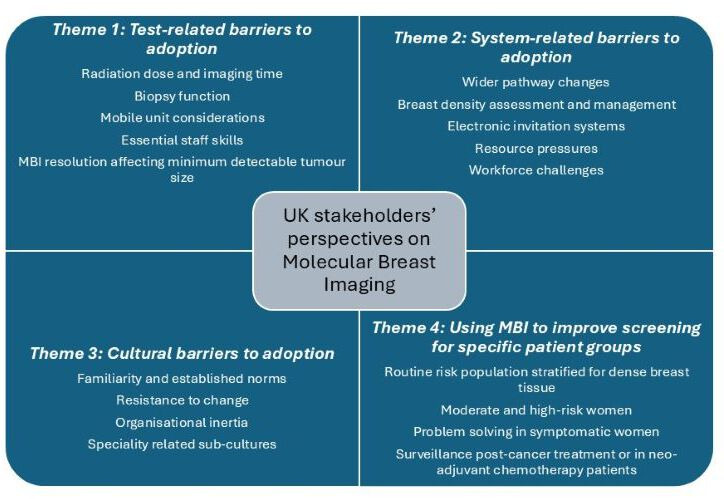
A diagrammatic representation of four themes identified in the analysis and the key components associated with each. MBI, molecular breast imaging.

Three types of barriers were identified to the adoption of MBI as a breast-imaging test: barriers related to the scan and scanning process, barriers related to the system, and broader cultural barriers that are present within the existing screening programme. Adoption of MBI into the screening programme is likely to require all three barriers to be addressed. The fourth theme explores how MBI could improve screening for different patient groups, provided that the outlined barriers are addressed. Figure 1 presents these four themes and the main components underpinning each.

### Theme 1: test-related barriers to adoption

Interviewees agreed unanimously that MBI could not replace mammography for women in the routine-risk screening programme due to scan time, radiation dose, scan cost, staff time and lower image resolution than mammography; “A number of things would hold it back from completely replacing it.” (S002, consultant clinical scientist in nuclear medicine).

When comparing the common whole-body effective dose of MBI with mammography (6.25 mSv and 0.5 mSv respectively), MBI is significantly higher (lower doses are being trialled in the USA). Participants consistently stated that dose needs to be reduced for MBI to be an acceptable imaging test in a screening environment, “(reducing) radiation doses would be great. It’s obviously really important.” (S012, third sector specialist breast care nurse). In addition, the current MBI scan time of 40 min was unanimously considered among frontline healthcare staff to be a barrier to adoption, “*…*a test that takes 40 minutes is just not feasible in a screening setting.” (S015, consultant breast radiologist).

Perceptions among different staff groups regarding radiation are an area of conflict within our data. Some breast radiologists expressed concerns about radiation levels: “it’s really difficult to see how it would become a screening tool because the radiation is too high” (S011, consultant breast radiologist). Others were concerned about patient attitudes: “women …I think would be …worried about the radiation dose of (MBI) even though, in medical terms, it’s negligible.” (S006, consultant breast radiologist). Nuclear medicine staff were less concerned: “I’m encouraged to see the dose (of MBI) being that low.” (S004, consultant nuclear radiologist) and “I think (patients) are not too concerned (about radiation exposure).” (S002, consultant clinical scientist in nuclear medicine).

Some marketed MBI systems do not offer biopsy functionality. If a lesion found on MBI was not visible on another imaging modality, a biopsy would be indicated to rule out malignancy. There was uncertainty in our data around whether MBI requires a biopsy function. Some participants thought that based on the MBI image lesions would likely be found, and therefore biopsied, on alternative imaging: “we’ve got ultrasound – ultrasound machines are getting better all the time; we’ve got extra views, we’ve got MRI” (S011, consultant breast radiologist). Others were concerned that, for a lesion visible on MBI but not on MRI or ultrasound, a biopsy function would be vital: “Actually, you need a biopsy tool …because you need to test it” (S003, senior breast radiographer).

The possibility of mobile scans was discussed with nuclear medicine staff with regulatory components to their roles, for example, radiation safety and Administration of Radioactive Substances Advisory Committee (ARSAC) licensing. One participant explained that, as with mobile positron emission tomography-computed tomography (PET-CT) scans, a mobile MBI scanner would require “an Environment Agency permit …under Environmental Permitting Regulations 2016, …consent from (the Health and Safety Executive) and the Ionising Radiations Regulations 2017 (Health and Safety Executive, 2017), (Care Quality Commission) approval for Ionising Radiation (Medical Exposures) Regulations 2017 (Department of Health and Social Care, 2017)” (S002, consultant clinical scientist in nuclear medicine). Significant resource implications arise from this legal framework; the same participant added that specialist staff would be required, including “a radiation waste advisor …radiation protection advisor, medical physics expert”, further increasing costs. Another participant explained that while PET-CT is considered ‘mobile’, there are limitations: “It’s mobile to the extent that it has to still go to the same sites that have ARSAC licence, site licences”, (S004, consultant nuclear medicine radiologist).

Given that MBI involves elements traditionally split between two specialisms, breast imaging and nuclear medicine, participants identified that cross-departmental skills are required. Nuclear medicine technologists specialise in administering radiation and undertaking scans which require specialist monitoring. Breast imaging technologists have advanced understanding of breast anatomy and positioning to maximise imaging coverage. Participants felt that early research into the performance of MBI machines should be located within nuclear medicine departments due to ARSAC regulations. Eventually, if adopted, MBI could be located in either department depending on local resources. Whichever department hosted, expertise relating to the other specialism will be required: “(You could) put it into another department …as long as you’ve got the right people working the machine that are trained.” (S004, consultant nuclear medicine radiologist).

The question of minimum resolution required for MBI to be clinically useful proved a difficult question to answer: “the size of the smallest invisible cancer is the answer (laughter), …we don’t want to miss cancer.” (S005, consultant breast radiologist). The same participant mentioned a threshold often used as a benchmark for MBI: “we have an arbitrary cut-off …of about 5 mm (for MRI)” (S005, consultant breast radiologist). Another participant mentioned the same cut-off but said that the grade of disease is also of interest: “Detecting small grade 1 cancers isn’t that important but detecting small grade 3 cancers is really important.” (S014, consultant breast radiologist). Another participant suggested a smaller cut-off: “the smallest lesion that we currently can detect on ultrasound …probably 3 mm” (S017, consultant breast radiologist). Ductal carcinoma in situ (DCIS), a non-invasive form of breast cancer, was raised by one participant, with concern that it may be missed with inadequate resolution: “we pick up DCIS, which hasn’t even got an invasive cancer to measure …(this) almost certainly makes a difference …I don’t think we can afford not to find those.” (S011, consultant breast radiologist).

### Theme 2: system-related barriers to adoption

System-level barriers centred on constraints within existing service structures. Participants highlighted issues such as wider pathway changes, breast density assessment, inadequate Information Technology (IT) systems and lack of resources, noting that these operational challenges would hinder adoption of MBI as a breast-imaging test.

Participants agreed that the current routine-risk NHSBSP pathway is likely to change soon, “There is a lot of movement in this space, and part of it is moving to that risk-stratified type of screening and the personalised.” (S016, third sector policy manager). However, uncertainty exists as to what these new pathways should look like. One suggestion was to shorten the screening round: “If you look at the interval cancers that turn up, most of them are turning up in the third year, so if we want to find more fast-growing cancers, the best thing to do is shorten our round length.” (S011, consultant breast radiologist). Another suggestion was to stratify screening using personalised risk assessment. Possible methods of risk assessment are much debated in the literature and in our data suggestions range from comprehensive assessments, such as in the Predicting the Risk of Breast Cancer at Screening (PROCAS) trial,^12^ to stratifying based on breast density, as in the Breast Screening - Risk Adaptive Imaging for Density (BRAID) trial.^4^ It was suggested that extra screening for those categorised as higher risk could be funded by less frequent screening for low-risk women: “If we’re looking at things from a purely cost effectiveness manner then the problem is some of the lower risk women wouldn’t meet the cost effectiveness threshold (for screening) …but it’s quite a difficult public health message and so I’m not sure whether we’ll move to that, but you can make quite a cogent argument for that.” (S019, consultant breast radiologist).

Breast density is not formally measured in the NHSBSP at present: “[We record] either normal or abnormal, and there’s no reference to density in (the) form that we fill in.” (S003, senior breast radiographer). Facilitating a screening programme stratified by density would require changes to workload: “it’s an extra click, and when you’ve only got one click anyway doubling the number of clicks you’ve got …may be too much.” (S005, consultant breast radiologist), and IT systems: “You have to retain the raw images. In terms of (image) storage, that is a massive undertaking” (S015, consultant breast radiologist). Breast density assessment would be required for most of the populations suggested for MBI, and most participants discussed measurement methods. Most would prefer a standardised, fully automated system of breast density measurement to limit impact on workload: “(radiologist assessment of breast density) would double the time for film reading and actually it’s dead easy to do as an automated method.” (S011, consultant breast radiologist). However, there was concern about whether currently existing fully automated tools are sufficiently well-developed: “I think a ‘to be determined’ automated method is going to be the way but I don’t think (current tools) are sophisticated enough.” (S006, consultant breast radiologist).

Electronic systems are currently in place to manage both mass screening invitations and imaging results. Our interviews suggested that current IT infrastructure will not support major change to NHSBSP pathways and that updates to systems are a national priority: “if you were to think about adding an additional part to the pathway that requires risk stratification …one of the major recommendations …one of (the government’s) pilot projects is developing a robust IT system for the breast screening programme.” (S016, third sector policy manager).

Insufficient NHS resources were cited widely as a barrier to introduction of a stratified screening programme. The term ‘resources’ covered many areas, such as staff, “there isn’t the staff to be able to deploy for an hour per patient for screening.” (S005, consultant breast radiologist); specialist skills, “you’d need trained …radiographers” (S004, consultant nuclear medicine radiologist); physical space, “availability of resources in terms of …clinical rooms” (S002, consultant clinical scientist in nuclear medicine); electronic invitation and stratification systems, “that somebody would have to manually (invite women at higher risk to a different/extra clinic) would be just horrendous …we would need some (electronic) flag or something to identify them (on the system)” (S021, senior breast radiographer); automated density assessment, “in terms of storage on PACS, (an automated density tool) is a massive undertaking. In most units, you just can’t do it.” (S015, consultant breast radiologist); and cost, “your breast unit is going to have to invest in a piece of kit” (S015, consultant breast radiologist).

Building on these resource and system-level challenges, participants described how such pressures made it difficult to generate momentum for change in overstretched departments with little to no dedicated research time for clinical staff, “When you’re up to your neck in alligators, it’s rather difficult to remember you’re trying to drain the swamp …(changes) will just take time, because we’ve got to keep doing the day job.” (S001, consultant clinical oncologist). Participants felt that these system pressures also limited the ability to build the necessary ‘clinical drive’ for implementation, “If clinicians are not interested, it’s just not going to happen.” (S002, consultant clinical scientist in nuclear medicine).

### Theme 3: cultural barriers to adoption

Cultural barriers were evident in how participants perceived existing screening processes. Their familiarity with existing approaches resulted in inertia and resistance to new techniques that extended beyond the system-related barriers, with participants commenting on how this is a broader issue than just breast cancer screening: “…the UK is not a flexible …health system. We can have things recommended, but it takes years, if not a decade, for things to be changed in a programme.” (S016, third sector policy manager). This inertia appeared to be linked to participants’ familiarity with existing imaging tests, suggesting that established practices and personal preferences may shape perspectives on the adoption of newer techniques: “I can’t envisage a situation where I would say, ‘…I’m not going to do an MRI. I’m going to do MBI instead,’ but then I love MRIs.” (S015, consultant breast radiologist). Other breast imaging tests, contrast-enhanced mammography (CEM) and digital breast tomosynthesis (DBT) are already used in some routine clinics: “we’re using DBT …for younger people, women under 50 as a problem-solving tool. We use (DBT) in our screening assessment clinics, women who are recalled through screening and also in our symptomatic clinics. With (CEM), we use it for problem solving and when we recall people back for assessment.” (S019, consultant breast radiologist). MBI is unlikely to be introduced in this way, with participants stating they would need evidence showing that MBI is diagnostically superior: “If it was only proven to be non-inferior to equivalent imaging modalities, that wouldn’t be enough…there’s already kit out there that it would be potentially competing against.” (S007, policy manager, Public Health England).

There were examples of subcultures among different specialties. Nuclear medicine specialists, who manage risks associated with radiopharmaceuticals day-to-day, had fewer reservations about the risk of MBI-associated radiation than breast screening staff. Breast screening staff were more comfortable with DBT and CEM, reflecting in part that MBI typically involves a higher radiation dose than CEM: “(CEM) has a slightly higher radiation dose than standard mammogram but not in the league of (MBI).” (S017, consultant breast radiologist). CEM can be added as an option to some mammography systems, as can DBT, requiring only a software update. It was suggested that most breast units have access to MRI machines too, but also that MRI is oversubscribed: “MRI is relatively resource intensive. We’re limited to the amount of time we get (sic) on the MR(I) scanner.” (S018, consultant oncoplastic breast surgeon).

### Theme 4: using MBI to improve screening for specific patient groups

If some or all the barriers can be addressed, participants highlighted six patient groups that could benefit from imaging with MBI. These included women in the routine-risk screening programme with dense breast tissue, women at moderate or high risk, symptomatic patients, and those undergoing post-cancer surveillance or neo-adjuvant chemotherapy.

Participants suggested that MBI could be beneficial in subsets of women, “Maybe …adding it in as a supplement for women with dense breasts.” (S014, consultant breast radiologist). Interviewees estimated that roughly 8% of women have the highest category of breast density (BI-RADS category D) and 30%–40% into categories C and D combined, these estimates are consistent with published population-based studies.^3^ It was unclear which categories of breast density should be considered for supplemental screening, but interviewees hypothesised that MBI is likely to be cost effective only for patients with extremely dense tissue, “We’re unlikely to do online supplemental imaging for category C because that’s 40% of the population. The real problem …is BI-RADS D.” (S019, consultant breast radiologist).

Moderate-risk patients, defined as having a lifetime risk of developing breast cancer of at least 17%–30%,^13^ are invited for screening with either mammography or MRI depending on age. These patients’ breast screening is managed by their local secondary care trust, not through the NHSBSP. It was suggested that MBI could be useful in these women, “There are lots of moderate or high-risk women not eligible for MRI. If those high-risk women had dense breasts as well, …I would want to use (MBI).” (S015, consultant breast radiologist). However, some participants had concerns, “I guess my issue for the higher-risk patients is that it’s the amount of radiation that they might be getting on an annual basis because those women will have annual screening.” (S017, consultant breast radiologist).

High-risk patients can be split into two groups. First, those with a lifetime breast cancer risk greater than 40%, whose screening is managed by the NHSBSP. Second, those with a lifetime risk of at least 30%, whose screening is managed locally according to National Institute for Health and Care Excellence (NICE) guidelines. A subset of these women was suggested, “The group of women …I worry about most are women who have a strong …family history of breast cancer, but we don’t identify a faulty gene …it doesn’t mean there’s not one there, just we can't find it. At the minute, they are only screened via mammography from the age of 40 …if mammograms were good enough, let’s say, from 35 onwards, I get the impression that’s probably a group of women that guidelines might say let’s start at 35 but, probably for costs reasons and availability of MRI, we withhold screening from that group of women.” (S010, consultant clinical geneticist).

Women presenting to primary care with symptoms are referred to hospital for clinical assessment, imaging and possible biopsy. Interviewees suggested that MBI could be a cheaper, less resource-intensive alternative to MRI in cases of discrepancy between mammogram and ultrasound. “I think it could potentially be a problem-solving tool for indeterminate lesions.” (S005, consultant breast radiologist).

Following curative breast cancer treatment, women are imaged using mammography annually for 5 years, until age 50 when screening is resumed by the NHSBSP. They could be considered for supplemental MBI in the incidence of dense breasts on mammography. “There aren’t tremendously tight guidelines around the surveillance follow-up of mammographically occult disease, so I think that’s why it’s an area of need.” (S018, consultant oncoplastic breast surgeon).

There was a suggestion that MBI could be used for monitoring tumours in patients receiving neo-adjuvant chemotherapy, “people are getting excited in imaging at the moment looking at early response to neoadjuvant chemotherapy.” (S018, consultant oncoplastic breast surgeon).

## Discussion

This study is the first to examine UK healthcare professionals’ perspectives on the feasibility of adopting MBI into breast cancer screening pathways. Since the interviews were conducted in 2020–2021, additional evidence on the diagnostic performance of MBI has been published, including a synthesis of performance outcomes^14^ and the multicentre Density MATTERS Trial.^15^ These developments provide broader context but do not alter the relevance of the perspectives captured in this study.

There is work ongoing in the USA trialling lower dose and time imaging protocols to improve MBI acceptability.^9 16^ We found that UK stakeholders would also require lower dose and time imaging protocols, as well as competitive diagnostic accuracy. Reduction of radiation dose and scan time, which are inversely related, should be prioritised in future development. Since data collection, evidence supporting MBI performance has broadened,^14 15^ although the modality has not yet been compared against alternatives such as CEM or abbreviated MRI (aMRI) in a randomised controlled trial.

Appropriate cross-departmental skills are required to deliver MBI, creating logistical barriers. However, a precedent exists for radioactive seed localisation.^17^ Specialist skills from both nuclear medicine and breast imaging are required. In the Mayo Clinic, MBI technologists act as ‘hybrid’ staff, but given NHS resource constraints, recruitment and training of such roles may be challenging. Local trusts are likely to decide where to locate MBI based on staffing, throughput and patient preference.^10^

Harms associated with cancer screening include overdiagnosis and false positives, leading to additional workload and patient anxiety.^18 19^ One study estimated that screening saves 5.7 lives per 1000 women but overdiagnoses 2.3 per 1000.^20^ Participants wished to understand how many extra cancers MBI might identify and what proportion may represent overdiagnosis. Our findings suggest that future prospective studies should consider surrogate measures such as tumour characteristics, interval cancer rates and cancer detection rates. This is supported by evidence suggesting these factors strongly influence tumour growth, detection and survival outcomes.^21^

Using MBI in BI-RADS D women would require large-scale stratification (~200 000 women annually), far greater than the current very-high-risk programme (<10 000).^22^ Interviewees strongly supported automated density assessment due to workforce pressures, as manual assessment was viewed as unfeasible.

Despite agreement that pathways need to change for women with dense breasts, staff portrayed lack of resources as a major barrier. Limited staff, IT, funding, space and equipment were repeatedly cited, consistent with earlier evidence describing such constraints as institutional barriers to change.^23^ NHS England^24^ has emphasised the need for robust, nationally managed IT systems and failures in current systems^25^ highlight the need for centrally organised electronic invitation management.

Generating sufficient evidence to support pathway change requires long-term research and health-economic evaluation, which can be resource-intensive and challenging. Participants described how familiarity with mammography, alongside workforce capacity pressures, may influence the adoption of new techniques. This perspective extended beyond breast imaging and was considered reflective of broader organisational patterns within the NHS. This aligns with wider literature suggesting that workload, limited support, and established systems can shape the pace at which evidence-based interventions are implemented. 26,29

Patients with BI-RADS D breast density have a fourfold increased lifetime risk of developing breast cancer.^3^ Questions are being asked nationally and internationally^30^,^32^ about the value of enhanced risk-stratified screening programmes for such patients. Viewing breast density as a risk factor requiring counselling raised further cultural and practical concerns, a point echoed by the findings from a UK National Screening Committee (UKNSC) risk-stratification workshop, where experts emphasised that shifting to population-level risk-based screening would require a careful, phased introduction.^33^ Participants highlighted that, unlike genetics clinics, breast-screening appointments do not have capacity for detailed shared decision-making around risk stratification. This is reflected in a systematic review of breast-cancer screening guidelines, which found that shared decision-making was rarely addressed outside guidance for women with a family history of breast cancer.^34^

We found strong evidence that MBI would not replace mammography, which remains effective for most women. A study of >40 000 women showed a 20% mortality reduction for those receiving biennial mammograms aged ≥55 at entry.^35^ Participants recognised this but identified subpopulations who might benefit from MBI. Although some raised concerns about radiation exposure highlighting the importance of demonstrating that any associated radiation dose is proportionate to clinical benefit, previous work shows that women are willing to accept MBI-associated radiation when making informed decisions.^10^

Wanders *et al*^3^ found that mammographic sensitivity decreases with increasing breast density, with women in the highest density categories also showing increased breast cancer risk and higher recall rates.^3^ Reflecting such evidence, the European Society Of Breast Imaging (EUSOBI) now recommends supplemental MRI for women with category D density.^5^ Patterson *et al* found handheld ultrasound (HHUS) was not cost effective in a UK setting.^36^ BRAID interim results reported higher cancer detection with automated breast ultrasound (ABUS), abbreviated MRI and CEM compared with mammography alone.^4^ In a separate USA cohort, adding MBI increased cancer detection from 3.2 to 12.0 per 1000 women screened.^9^ These figures are descriptive only due to cross study differences but highlight potential value. Other studies suggest MBI has promising sensitivity and specificity in dense breasts,^1537^,^39^ and US cost-effectiveness analysis suggests MBI may reduce cost per cancer detected.^40^ Further UK-specific evidence is required.

Another potential group is patients undergoing neo-adjuvant chemotherapy. A small prospective study suggested MBI may have a role in monitoring, but larger studies are needed.^41^

## Conclusion

MBI has potential benefits for targeted groups, but meaningful adoption will require technical refinements, stronger UK evidence and solutions to workforce and system constraints.

## Supplementary material

10.1136/bmjopen-2025-113676 online supplemental file 1

## Data Availability

Data are available upon reasonable request.
