# Supplementary material for: Is molecular breast imaging suitable for use in UK breast cancer pathways? A qualitative study exploring healthcare professionals’ perspectives
Source: BMJ Open. 2026 Jun 3;16(6):e113676. doi: 10.1136/bmjopen-2025-113676 (PMC13239368; doi:10.1136/bmjopen-2025-113676)
Supplement: online supplemental file 1 [file bmjopen-16-6-s001.docx]

LDMBI staff topic guide - interview

Questions may be skipped if interviewer feels that they have been addressed earlier in the interview.

# Introduction

- Prior to starting the recording, informed consent to be taken unless already done so prior to the date of this interview/focus group.
- Confidentiality statement to be re-iterated: ‘Individual responses may be described in research outputs, however all possible precautions will be taken to disguise individuals’ identities so that readers of any such output will be unable to link you to this study’
- Let interviewee know that recording is about to begin and start the recording.
- Thanks for taking the time to participate in this study [We have around an hour to complete the interview today].
- First, let’s do the admin, then I will introduce you to who we are and what we do and then we can get started with the interview questions. Does this sound ok to you?
  - Admin
    - Have you received the study documents?
      - Consent form: happy to be audio recorded? [Your contribution to this study will be completely anonymised] Go through each point and sign/date form then give to interviewer.
  - Who we are
    - We work for Newcastle Hospitals NHS Foundation Trust, in collaboration with Newcastle University and the Newcastle MIC team (MedTech In Vitro Diagnostics), who specialise in the evaluation of diagnostic tests and devices.
    - We are working in collaboration with the company who are working on developing this technology but we are independent evaluators employed by the NHS.
  - In this study, we are performing a set of interviews and focus groups with key stakeholders to understand what the current practice is for screening breast cancer in the UK and to explore how this pathway could be altered with the introduction of this new technique.
    - The plan is to ask you some questions about the current pathway for breast cancer screening
    - Then ask you to look at the video describing the new test, which we will then discuss.
  - Does this all make sense? Do you have any questions for us so far?[happy to proceed]

**Key**

Black – All staff

Pink – Breast Screening staff

Blue – Nuclear Medicine staff

# Interviewee details

- Where do you work and what is your job role?
- Length of time in role, previous relevant roles.
- Estimate size and nature of the service they work in (catchment area, number of tests, etc).

# Current care pathway

- The first thing I’d like us to talk about is the breast cancer screening pathway [show document].
- This is a draft of the pathway that we think a woman in the National Breast Cancer Screening pathway will follow. It is only in draft form and has been developed on the basis of conversations with clinicians that we collaborate with and as a result of clinical shadowing.
- What we would like you to do is work your way through this pathway, from top to bottom, box by box, to see if you think it is representative of your current practice.
- Also, if you could talk us through your thinking at each stage that would be very useful for the recording.
- Alongside this, we might interrupt you from time to time, to ask specific questions we have.

## Accuracy of draft pathway

- Go through each part of the draft pathway.
- Document any omissions/inaccuracies in draft care pathway.
- Need to understand more accurately where MRI fits within this pathway.

## Issues with current pathway

- What works well and what doesn’t.

# Introduce low dose molecular breast imaging

## About the technique

- Introduction to (LD)MBI concept/product
  - Poor sensitivity of mammography to tumours in dense breast tissue.
  - Potential alternative, MBI, which is a nuclear medicine technique not reliant on a density differential. MBI uses a short-lived radiotracer, injected into the arm of the patient, which is attracted to any areas of cancer inside the breast. With cancer cells being typically more metabolically active than normal cells, they take up more of this tracer, and show ‘hot’ on the subsequent scan image.
  - Show image of gantry in use in the USA.
  - Show some imaging examples of mammo vs MBI images from the literature.
  - Present diagnostic accuracy reported in literature, acknowledging quality issues and biases.

# Delivery of the procedure

- In terms of delivering the procedure, not yet thinking about the adoption process, we have broken it down into the following elements:
  - Production of radiopharmaceutical
  - Administration of radiopharmaceutical (injection – unsealed source)
  - Taking the imaging
  - Interpretation/reporting
- Are there any other elements to consider?
- Which of these could/should take place within a nuclear medicine setting, and which within breast screening?
- Are there any other comparable procedures where these elements are split across settings?
- Currently in the US, patients are advised to **fast**, come for the scan on a certain day of their **menstrual cycle** and are **kept warm during the scan**. Do you know why this might be advised? Are there any other practicalities which need to be undertaken?
- Would this be deliverable as a **mobile service**?

# Discussion

1. Could you outline the process for adoption of a new nuclear medicine technique in general?
   - Give the example if necessary of adoption of radioactive seed localisation for breast cancer before surgery.
2. Do you see a place for LDMBI in the breast screening pathway? If so, where?
   - initial screening? second line screening? patient subgroups e.g. general screening versus higher risk groups? Implants? Large breasts? Post cancer? Dense breasts?
   - In which of the above groups would LDMBI be particularly useful?
   - From your experience can you estimate the proportion of women with dense breasts? Do you think density affects decisions? What do you think is the best way to measure density? Would it be a burden to estimate density on screening mammograms? Are you trained to do this?
3. Are there clinical scenarios where this technology would alter your decision making?
   - How would it alter your decision making?
4. What do you perceive to be the advantages of this technology?
   - patient outcomes, costs, efficiency, etc
5. What do you see as the barriers to adopting the technology into the pathway?
   - Patient flow and timing, patient experience and compliance, image acquisition and interpretation time, costs, staffing, nature and quality of images.
   - Regulations, training, staffing
   - Are there solutions to these barriers?
6. What are the practical changes that would need to be made to accommodate LDMBI
   - Resources, training, facilities, etc
7. Would you have a role in the implementation of pathway changes? If yes, what would this role be?
8. Given your knowledge of existing evidence and guidelines, why do you think that (LD)MBI hasn’t been introduced into the breast screening pathway already?
9. What evidence on the technique would you want to see in order to be confident about adopting LDMBI in to the current care pathway? If you could design the perfect clinical trial to generate this evidence, what would it look like?
   1. Thinking about the PICO model for framing this research question with a view to designing a clinical trial:
      1. Population - Which patient population would you like to see this trial in? Eg all screening age women, dense breast tissue in screening age, young, high risk etc?
      2. Index test – LDMBI
      3. Comparison - What is the ‘gold standard test’ to compare MBI with?
      4. Outcome – Main question plus secondary outcomes ?long-term outcomes
10. What would be the pathway to adoption if LDMBI was to be introduced as first line screening and as second line screening? Or perhaps it would fit better in the symptomatic pathway?

# Stakeholders (snowball sampling)

- Can you suggest who we might need to speak to next to improve our knowledge of this subject?

# AOB

- Is there anything that we haven’t discussed that you think is important to the project?
