# Supplementary material for: Is molecular breast imaging suitable for use in UK breast cancer pathways? A qualitative study exploring healthcare professionals’ perspectives
Source: BMJ Open. 2026 Jun 3;16(6):e113676. doi: 10.1136/bmjopen-2025-113676 (PMC13239368; doi:10.1136/bmjopen-2025-113676)
Supplement: Supplementary file 2 [file bmjopen-2025-113676.reviewer_comments.pdf]

PEER REVIEW HISTORY

BMJ Open publishes all reviews undertaken for accepted manuscripts. Reviewers are asked to complete a checklist review form and are provided with free text boxes to elaborate on their assessment. These free text comments are reproduced below.

ARTICLE DETAILS

Title (Provisional)

Is Molecular Breast Imaging suitable for use in UK breast cancer pathways? A qualitative study exploring healthcare professionals' perspectives.

Authors

Elliott, Helen; Allen, A. Joy; Forester, Nerys D; Graziadio, Sara; Jones, William Stephen; Lendrem, Clare; Pearce, Mark; Powell, Timothy; Bray, Alison; Scott, Jason

---

VERSION 1 - REVIEW

**Reviewer**	**1**
**Name**	**Starkings, Rachel**
**Affiliation**	**Brighton and Sussex Medical School, SHORE-C**
**Date**	**12-Jan-2026**
**COI**	**None**

This is a well written paper with distinct purpose and outcome. I only had a few comments:

The limitations are included in the abstract but not in the body of the paper, please include.

I think diagram 1 is missing the label of theme 3. It may be clearer to lay this out with more distinction.

Could you please include your topic guide? This may be linked to the other manuscript, but I wasn't clear on that.

Can you clarify a bit further as to why you used 2 interviewers?

**Reviewer**	**2**
**Name**	**Neven, P.**
**Affiliation**	**Univ Hosp Leuven, Multidiscplinary Breast Center**
**Date**	**02-Feb-2026**
**COI**	**None**

The 'research' question the authors do ask is relevant and interesting. They explore 22 health care professionals' perspectives on the introduction of moleculair breast imaging mainly for screening purposes in the UK. The authors previously published already on the women's perspectives of molecular breast imaging. (Is there a difference in reference 10 and reference 27?)

My major concern is that 1. the manuscript is very (too) long to read 2. the perspectives (questions on validity of and clinical utility of this new technique) should be more organized and better grouped to make the story easier to understand and 3. there is a long time between the research was performed (October 2021 and today, Feb 2026) also requiring to update the out-dated references. How up-to-date was the structured information about MBI including sensitivity and specificity of the technique? They refer several times to the work of Hruska et al. but don't mention the most recent work regarding 'Density MATTERS Trial'. Data regarding the diagnostic performance of MBI for supplemental breast cancer screening in high density breasts has thus far been limited to single-institutional studies (Smith KA, et al.Molecular Breast Imaging in the Screening Setting. J Breast Imaging 2023;5(3):240–247). Hruska C, et al. recently published the Density MATTERS Trial Molecular Breast Imaging and Digital Breast Tomosynthesis for Dense Breast Screening: The Density MATTERS Trial. Radiology 2025;316(3):e243953.

The authors should also refer to implementation of artifical intelligence and abbreviated breast MR imaging which has improved screening dense breasts (BRAID trial which they refer to). Also, breast cancer is a heterogeneous disease and early detection might differ between breast cancer subtypes using the different MBI techniques that have been used.A major concern is that MBI was never tested in a randomized controlled trial against CEM which also in 2026 leads to uncertainty using this technique.

Overall, the information is usefull but please update references!

**Reviewer**	**3**
**Name**	**Sebri , Valeria**
**Affiliation**	**European Institute of Oncology IRCCS Library**
**Date**	**04-Mar-2026**
**COI**	**None**

Dear authors,

thank you for your contribution. Here are my suggestions

- Introduction: please, foster your background with further details regarding psychological constructs related to breast cancer (Please, see: Sebri et al., 2020; 2023.) and related

interventions. For example, what about how to address these points? Second, please, structure your hypothesis and goals better

- Methods: the recruitment process should be better explained

- Discussion: I would suggest proposing new approaches to this topic. As known, new technologies are currently an innovative, effective approach that could be applied, with benefits and limits. Please, discuss this point, considering your research study and findings (see: Triberti et al., 2021).

References

- Triberti, S., Petrella, F., Gorini, A., Pappalardo, O., Sebri, V., Savioni, L., ... & Pravettoni, G. (2021). Augmenting surgery: medical students' assessment and ergonomics of 3d holograms vs. CT scans for pre-operative planning. EAI Endorsed Transactions on Pervasive Health and Technology, 7(25).

- Sebri, V., Savioni, L., Triberti, S., Durosini, I., Mazzocco, K., & Pravettoni, G. (2020). Do you transfer your skills? From sports to health management in cancer patients. Frontiers in Psychology, 11, 546.

- Sebri, V., Durosini, I., Strika, M., Pizzoli, S. F. M., Mazzocco, K., & Pravettoni, G. (2023). Virtual reality for the promotion of interoception awareness and body image in breast cancer survivors: a study protocol. Frontiers in Psychology, 14, 1165905.

VERSION 1 - AUTHOR RESPONSE

Reviewer 1, Dr. Rachel Starkings, Brighton and Sussex Medical School

1. *The limitations are included in the abstract but not in the body of the paper, please include.*

   Response: Limitations have been included in the body of the paper, in the methods section.

2. *I think diagram 1 is missing the label of theme 3. It may be clearer to lay this out with more distinction.*

   Response: Themes have been restructured, please see the response to reviewer 2, comment 3.

3. *Could you please include your topic guide? This may be linked to the other manuscript, but I wasn't clear on that.*

   Response: We have included the first version of the topic guide as supplementary material.

4. *Can you clarify a bit further as to why you used 2 interviewers?*

   Response: We have added further explanation in the methods section to clarify that two interviewers were used to enhance transparency, reflexivity and completeness of data collection. We have acknowledged that the presence of two researchers may have influenced how comfortable some participants felt.

Reviewer 2, Dr. P. Neven, Univ Hosp Leuven

1. *The 'research' question the authors do ask is relevant and interesting. They explore 22 health care professionals' perspectives on the introduction of molecular breast imaging mainly for screening purposes in the UK. The authors previously published already on the women's perspectives of molecular breast imaging. (Is there a difference in reference 10 and reference 27?)*

   Response: This duplicate reference has been removed.

2. *The manuscript is very (too) long to read.*

   Response: We have reduced the word count from 5139 to 4625.

3. *The perspectives (questions on validity of and clinical utility of this new technique) should be more organized and better grouped to make the story easier to understand.*

   Response: Thank you for this helpful suggestion. In response, we have restructured our results and discussion to describe test-specific, system-related and cultural barriers to adoption followed by participants suggestions for improving screening using MBI. The underlying qualitative analysis remains unchanged except for this restructure aimed at improving the narrative.

4. *There is a long time between the research was performed (October 2021 and today, Feb 2026) also requiring to update the out-dated references. How up-to-date was the structured information about MBI including sensitivity and specificity of the technique? They refer several times to the work of Hruska et al. but don't mention the most recent work regarding 'Density MATTERS Trial'. Data regarding the diagnostic performance of MBI for supplemental breast cancer screening in high density breasts has thus far been*

limited to single-institutional studies (Smith KA, et al.Molecular Breast Imaging in the Screening Setting. J Breast Imaging 2023;5(3):240–247). Hruska C, et al. recently published the Density MATTERS Trial Molecular Breast Imaging and Digital Breast Tomosynthesis for Dense Breast Screening: The Density MATTERS Trial. Radiology 2025;316(3):e243953.

Response: We agree that important new evidence on MBI has emerged since the interviews were conducted in 2021. The structured information provided to participants at the time was based on the best available evidence then, which primarily included early work by Hruska and colleagues. We have now clarified this in the methods section. We have updated the manuscript to include more recent data on diagnostic performance, including the 2023 summary by Smith et al. and findings from the Density MATTERS Trial published in 2025. These updated references are now incorporated into the discussion to ensure the manuscript reflects the current evidence base. We also added a statement and limitation acknowledging that participants' understanding was shaped by the evidence available at the time of interview, and that subsequent developments (including the Density MATTERS results) were not part of the information presented to them.

5. *The authors should also refer to implementation of artificial intelligence and abbreviated breast MR imaging which has improved screening dense breasts (BRAID trial which they refer to). Also, breast cancer is a heterogeneous disease and early detection might differ between breast cancer subtypes using the different MBI techniques that have been used. A major concern is that MBI was never tested in a randomized controlled trial against CEM which also in 2026 leads to uncertainty using this technique.*

   Response: We also agree that the lack of RCTs comparing MBI with CEM or abbreviated MRI is a major limitation of MBI, and we've emphasised this in the manuscript. While AI is a developing area in breast imaging, it's mainly being used for mammographic density assessment and mammogram reading. There are no validated AI tools for interpreting MBI yet, so we don't think it's directly relevant to evaluating MBI at this stage. Finally, current MBI studies haven't been designed or powered to look at detection by molecular subtype, and there's no consistent evidence showing subtype-specific differences between MBI techniques.

6. *Please update references!*

   Response: References have been updates in line with your suggestions.

Reviewer 3, Dr. Valeria Sebri, European Institute of Oncology IRCCS Library

1. *Introduction: please, foster your background with further details regarding psychological constructs related to breast cancer (Please, see Sebri et al., 2020: "Do you transfer your skills? From sports to health management in cancer patients", and Sebri et al., 2023: "Virtual reality for the promotion of interoception awareness and body image in breast cancer survivors: a study protocol") and related interventions. For example, what about how to address these points?*

   Response: We have not included the two papers cited by the reviewer as they relate specifically to survivorship related constructs, including body-image concerns and skills-transfer mechanisms for coping with a cancer diagnosis. We have reviewed them carefully; however, we do not feel they are relevant to our manuscript, which focuses on stakeholder perceptions of MBI as a potential tool within the UK breast screening programme. Individuals undergoing screening have not received, and may never receive, a breast cancer diagnosis, and therefore survivorship focused psychological constructs fall outside the scope of our focus. We remain open to reconsidering if the reviewer still considers a link.

2. *Introduction: please, structure your hypothesis and goals better.*

   Response: Because this was an exploratory qualitative study, no hypothesis was specified a priori. We have revised the introduction to present the study aim more clearly, in line with qualitative research conventions.

3. *Methods: the recruitment process should be better explained.*

   Response: Addressed as per the editor's comments 2, 3, 4, 5, 6.

4. *Discussion: I would suggest proposing new approaches to this topic. As known, new technologies are currently an innovative, effective approach that could be applied, with benefits and limits. Please, discuss this point, considering your research study and findings (see: Triberti et al., 2021: Augmenting surgery: medical students' assessment and ergonomics of 3d holograms vs. CT scans for pre-operative planning.).*

   Response: Discussion of future research and new approaches are included in lines 387-389, 400-403, 449, and 451-452. Similarly to our response to comment 1, we have reviewed the cited work, and it falls outside the scope of our focus.
